# Chloroform-Assisted Phenol Extraction Improving Proteome Profiling of Maize Embryos through Selective Depletion of High-Abundance Storage Proteins

**DOI:** 10.1371/journal.pone.0112724

**Published:** 2014-11-11

**Authors:** Erhui Xiong, Xiaolin Wu, Le Yang, Fangping Gong, Fuju Tai, Wei Wang

**Affiliations:** Collaborative Innovation Center of Henan Grain Crops, College of Life Science, Henan Agricultural University, Zhengzhou 450002, China; Università della Calabria, Italy

## Abstract

The presence of abundant storage proteins in plant embryos greatly impedes seed proteomics analysis. Vicilin (or globulin-1) is the most abundant storage protein in maize embryo. There is a need to deplete the vicilins from maize embryo extracts for enhanced proteomics analysis. We here reported a chloroform-assisted phenol extraction (CAPE) method for vicilin depletion. By CAPE, maize embryo proteins were first extracted in an aqueous buffer, denatured by chloroform and then subjected to phenol extraction. We found that CAPE can effectively deplete the vicilins from maize embryo extract, allowing the detection of low-abundance proteins that were masked by vicilins in 2-DE gel. The novelty of CAPE is that it selectively depletes abundant storage proteins from embryo extracts of both monocot (maize) and dicot (soybean and pea) seeds, whereas other embryo proteins were not depleted. CAPE can significantly improve proteome profiling of embryos and extends the application of chloroform and phenol extraction in plant proteomics. In addition, the rationale behind CAPE depletion of abundant storage proteins was explored.

## Introduction

Maize (*Zea mays* L.) is one of the most important cereal crops worldwide [1]. For a long time, maize has been a staple food of the world's population and a primary nutrient source for animal feed. In recent years, maize has been used for biofuel production [2]. As one of the most frequently harvested organs in agriculture, maize seed contains about 10% proteins, in which 60–80% are storage proteins, mainly existing in embryo as nutrient reservoir for seed germination and early seedling establishment. Likewise, maize embryo is very important for human and livestock nutrition due to its high contents of protein and oil [3].

In maize embryo, vicilin (or globulin-1) is the most abundant, basic (arginine-rich) storage protein. Typically, vicilins are sparsely glycosylated trimeric clusters and each subunit contains two conserved cupin domains, characteristic of Cupin_2 globulin superfamily [4]–[6]. Maize vicilin is encoded by a single but polymorphic *Glb1* gene [7]–[8]. The expression of *Glb1* is embryo specific [7] and regulated by ABA [9]. Recently, maize vicilin was identified as a novel allergen [1]. Due to its high abundance and composition complexity, maize vicilins impede embryo proteomics analysis to a great extent.

Depleting high abundance proteins is often an essential step in enhanced proteomics of complex samples, e.g. depleting storage proteins from legume seeds [10], RuBisCO from leaf extract [11], agglutinin from *Pinellia ternata* tuber extract [12] and albumin and IgG from serum [13]–[14]. Currently, no methods have been reported to deplete vicilins or abundant storage proteins from maize embryo extracts. We reported here a chloroform-assisted phenol extraction (CAPE) method for vicilin depletion from maize embryo extracts. CAPE is also effective for depletion of abundant storage proteins in dicot (soybean and pea) seeds.

## Materials and Methods

### Plant material and sampling

Maize (*Zea mays* L. cv. Zhengdan 958), soybean (*Glycine max* L.) and pea (*Pisum sativum* L.) seeds were bought from Henan Qiule Seed Industry Science & Technology Co. Ltd (Zhengzhou, China). Mature maize seed consists of three genetically distinct components: embryo, endosperm and coat. The embryo is the young organism before it emerges from the seed. Dry maize seeds were soaked in water for 2 h to soften starchy endosperm. Then, the embryos were manually took out, rinsed and used for protein extraction. Likewise, soybean and pea seeds were soaked in water for 2 h to remove seed coat and whole embryos were used for protein extraction.

### Reagents

All chemicals used were of analytical grade. High purity deionized water (18 MO.cm) was used throughout the experiment. Chloroform, buffered phenol (pH 8.0) and a cocktail of protease inhibitors were purchased from Sigma-Aldrich Co. LLC (St. Louis, MO, USA). Electrophoresis reagents and IPG strips were obtained from GE Healthcare Life Sciences (Pittsburgh, PA, USA).

### CAPE

**Figure 1:**
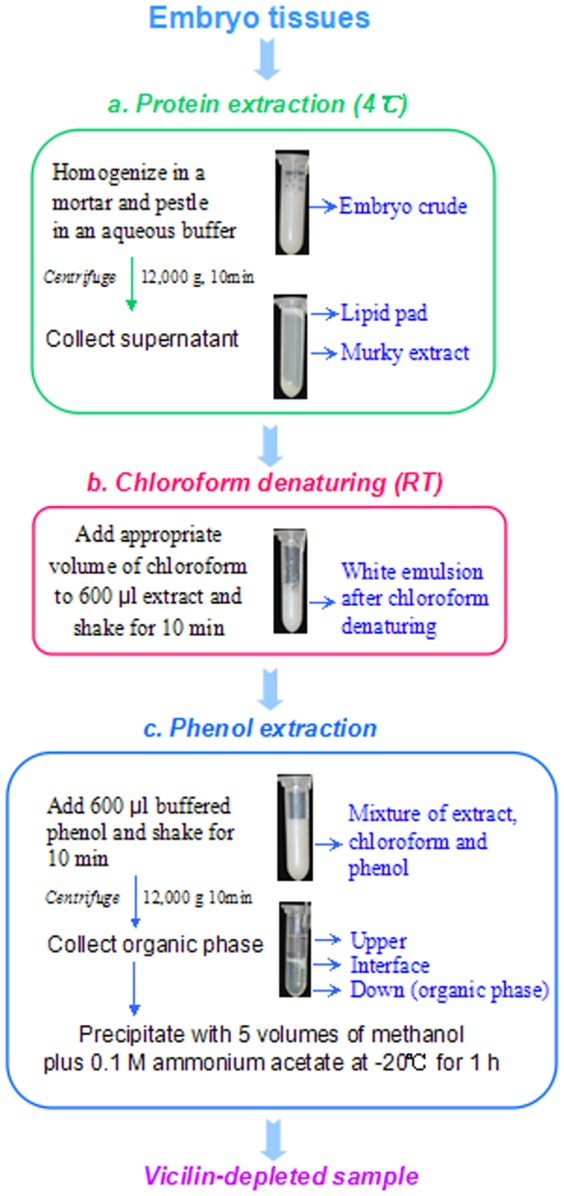
Schematic overview of the CAPE protocol. It was demonstrated to process 600 µl maize embryo extract in 2.0 ml Eppendorf tube.

The CAPE protocol includes three parts (**Figure 1**). It is designed for 600 µl embryo extract to be processed in 2.0 ml Eppendorf tube and can be scaled up for bigger volumes.

#### a. Protein extraction

Embryo tissues (0.1 g fresh weight) were homogenized in a cold mortar in 1.0 ml of buffer containing 0.25 M Tris-HCl (pH 7.5), 1% SDS, 14 mM DTT and a cocktail of protease inhibitors (4°C). *Note: for embryo from developing, young seeds, less extract buffer (e.g. 0.1 g versus 0.5 ml) can be used*. The homogenate was centrifuged at 12,000 g for 10 min (4°C). The resulting supernatant was still murky with a white solid layer floating on top, indicating the presence of large amount of lipids. The supernatant (embryo extract) was centrifuged again as above and subjected to chloroform denaturing.

As comparisons, aliquots of the embryo extract were mixed with equal volume of chloroform or buffered phenol (pH 8.0). After phase separation by centrifugation, proteins in each parts of the mixture were analyzed using SDS-PAGE.

#### b. Chloroform denaturing

Transfer 600 µl of the embryo extract to each 2.0 ml Eppendorf tube and add chloroform at a volume ratio of 1∶0 (extract/chloroform), 1∶0.25, 1∶0.33, 1∶0.5 and 1∶1, corresponding to 0, 150, 200, 300 and 600 µl chloroform, respectively. The mixture was thoroughly mixed by shaking for 10 min at room temperature (RT). *Note: Chloroform denaturing can be extended to 2 h as a pause point*. In the resulting white emulsion, embryo proteins were violently denatured by chloroform. The emulsion, without centrifugation, was directly subjected to phenol extraction.

#### c. Phenol extraction

Add 600 µl of buffered phenol (pH 8.0) to each tube containing the chloroform-treated extracts and thoroughly shook for 10 min at RT. *Note: In the presence of chloroform, organic phase was more hydrophobic and heavier, resulting in a quick and clear phase separation, with the organic phase (containing proteins) in the bottom*. After phase separation by centrifugation, the organic phase was transferred to new 2.0 ml Eppendorf tubes (300 µl each) and precipitated with 5 volumes of methanol containing 0.1 M ammonium acetate for 1 h (−20°C).

Proteins were precipitated by centrifugation at 15,000 g for 10 min (4°C) and washed with cold acetone and 80% cold acetone each once. Air-dried proteins were dissolved in a buffer of choice. Protein content was determined using Bio-Rad Protein Assay Kit and bovine serum albumin as a standard.

### SDS-PAGE

SDS-PAGE was performed using 13.5% resolving and 5% stacking polyacrylamide gels in a Bio-Rad system (Bio-Rad, Hercules, CA, USA) using the Laemmli buffers (Laemmli 1976). The gels were stained with Coomassie brilliant blue (CBB) R overnight and destained in 7% acetic acid until a clear background.

### Two dimensional gel electrophoresis (2-DE)

Isoelectric focusing (IEF) was performed using 11-cm linear pH 4–7 IPG strips with the Ettan III system (GE Healthcare, USA). About 600 µg proteins were loaded into the strip by passive rehydration overnight at RT. The IEF voltage was set at 250 V for 1 h, 1,000 V for 4 h, finally increasing to 8,000 V for 4 h, and holding for 10 h (20°C). Focused strips were equilibrated in Buffer I (0.1 M Tris–HCl, pH 8.8, 2% SDS, 6 M urea, 30% glycerol, 0.1 M DTT) and then in Buffer II (same as Buffer I, but with 0.25 M iodoacetamide instead of DTT) for 15 min each. SDS-PAGE was run on a 13.5% gel with 0.1% SDS in the gel and the running buffer. The gels were stained with 0.1% CBB G-250 overnight and destained in 7% acetic acid until a clear background. Digital images of the gels were processed using PDQUEST software (Bio-Rad).

### MS/MS and protein identification

Protein spots of interest were reduced (10 mM DTT), alkylated (50 mM iodoacetic acid), and then digested with 20 ng/µl trypsin for 16 h at 37°C in 50 mM ammonium bicarbonate. The supernatants were vacuum-dried and dissolved in 10 µl of 0.1% trifluoroacetic acid, and 0.5 µl of the solution was added onto a matrix consisting of 0.5 µl of 5 mg/ml 2, 5-dihydroxybenzoic acid in water: acetonitrile (2∶1). The digested fragments were analyzed using a MALDI-TOF/TOF analyzer (ultraflex III, Bruker, Germany). MS/MS spectra were acquired in the positive ion mode and automatically submitted to Mascot 2.2 (http://www.matrixscience.com) for peptide mass finger printing against the NCBInr 20140323 database (38032689 sequences). The taxonomy used was Viridiplantae (green plants) (1749470 sequences). The search parameters were as follows: type of search: MALDI-TOF ion search; enzyme: trypsin; fixed modifications: carbamidomethyl (C); variable modifications: acetyl (N-terminal) and oxidation (M); mass values: monoisotopic; protein mass: unrestricted; peptide mass tolerance: ±100 ppm; fragment mass tolerance: ±0.5 Da; max missed cleavages: 1; instrument type: MALDI-TOF-TOF. Only significant scores defined by Mascot probability analysis greater than “identity” were considered for assigning protein identity. All of the positive protein identification scores were significant (P<0.05, score>50).

## Results and Discussion

### Development of the CAPE protocol

**Figure 2:**
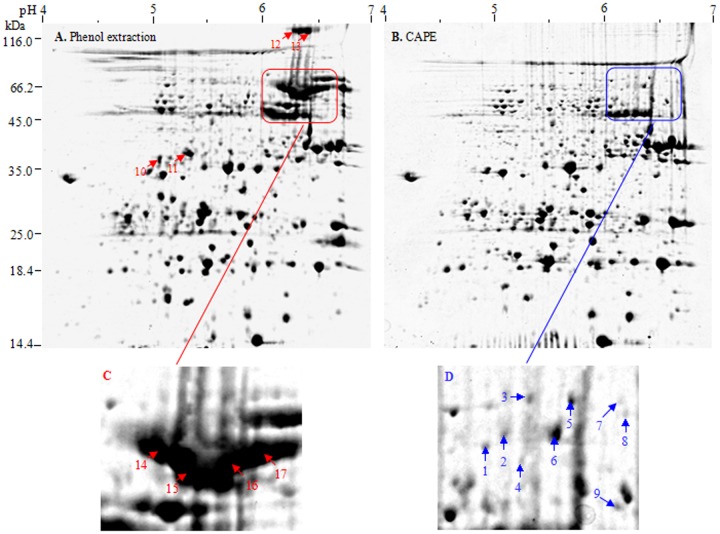
Depletion of maize vicilins by CAPE. **A**: Phenol extraction (control). **B**: CAPE. Maize embryo proteins (600 µg) were separated by IEF with 11-cm linear pH 4–7 IPG strips and then by SDS-PAGE. The magnified regions indicate before and after vicilin depletion.

Currently, methods based on trichloroacetic acid (TCA)/acetone precipitation or phenol extraction are widely used in plant proteomics [15]–[16]. Most recently, we described in detail sample preparation method integrating TCA/acetone precipitation and phenol extraction for crop proteomics [15]. However, 2-DE image of maize embryo, based on these reported available protocols, was dominated by high-abundance vicilins (**Figure 2**), which inevitably masked low-abundance proteins with similar p*I*s and sizes and impeded to dig deep into embryo proteome. In preliminary experiments, we tried many protocols, but all failed to effectively deplete vicilins from maize embryo extracts. Fortunately, we found that chloroform denaturing prior to phenol extraction can effectively and selectively deplete vicilins from maize embryo extract.

**Figure 3:**
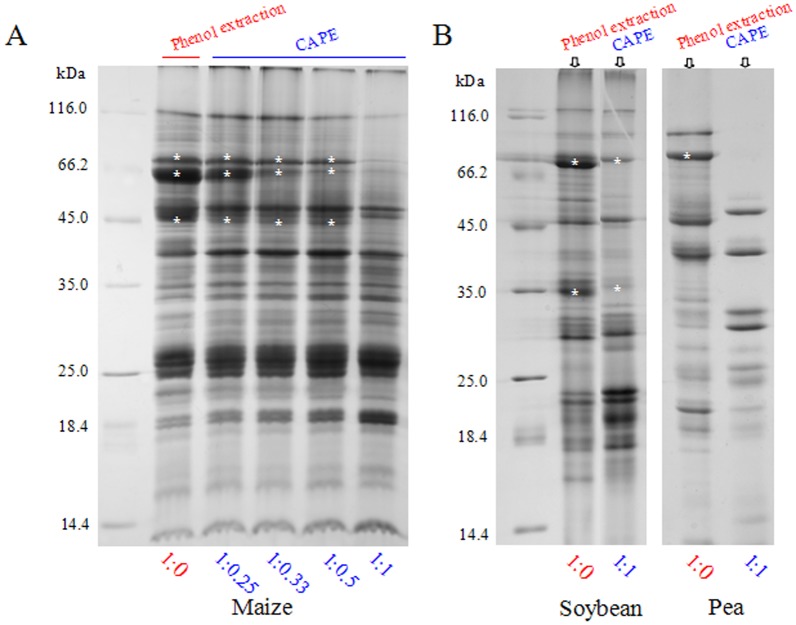
Optimization of chloroform ratio used in CAPE. Embryo extracts were mixed well with chloroform at different ratios. Proteins obtained from CAPE and phenol extraction (control) were compared by SDS-PAGE. The asterisks (*) indicated vicilins or abundant storage proteins.

We first optimized the CAPE method regarding the amount and treatment time of chloroform before phenol extraction. A ratio 1∶1 (v∶v) of extract/chloroform was found to be sufficient to deplete vicilins from maize embryo extract, as in the case of soybean and pea seeds (**Figure 3**). As demonstrated by SDS-PAGE, major vicilin (or abundant storage protein) bands (indicated by asterisk) decreased continually in abundance with the increase of chloroform amount. No significant difference of incubation time of chloroform denaturing was observed between 10–30 min. Thus, a ratio of 1∶1 (extract/chloroform) and 10 min incubation in CAPE were used in subsequent experiments. For dilute embryo extract, a 1∶0.5 (extract/chloroform) was sufficient.

**Figure 4:**
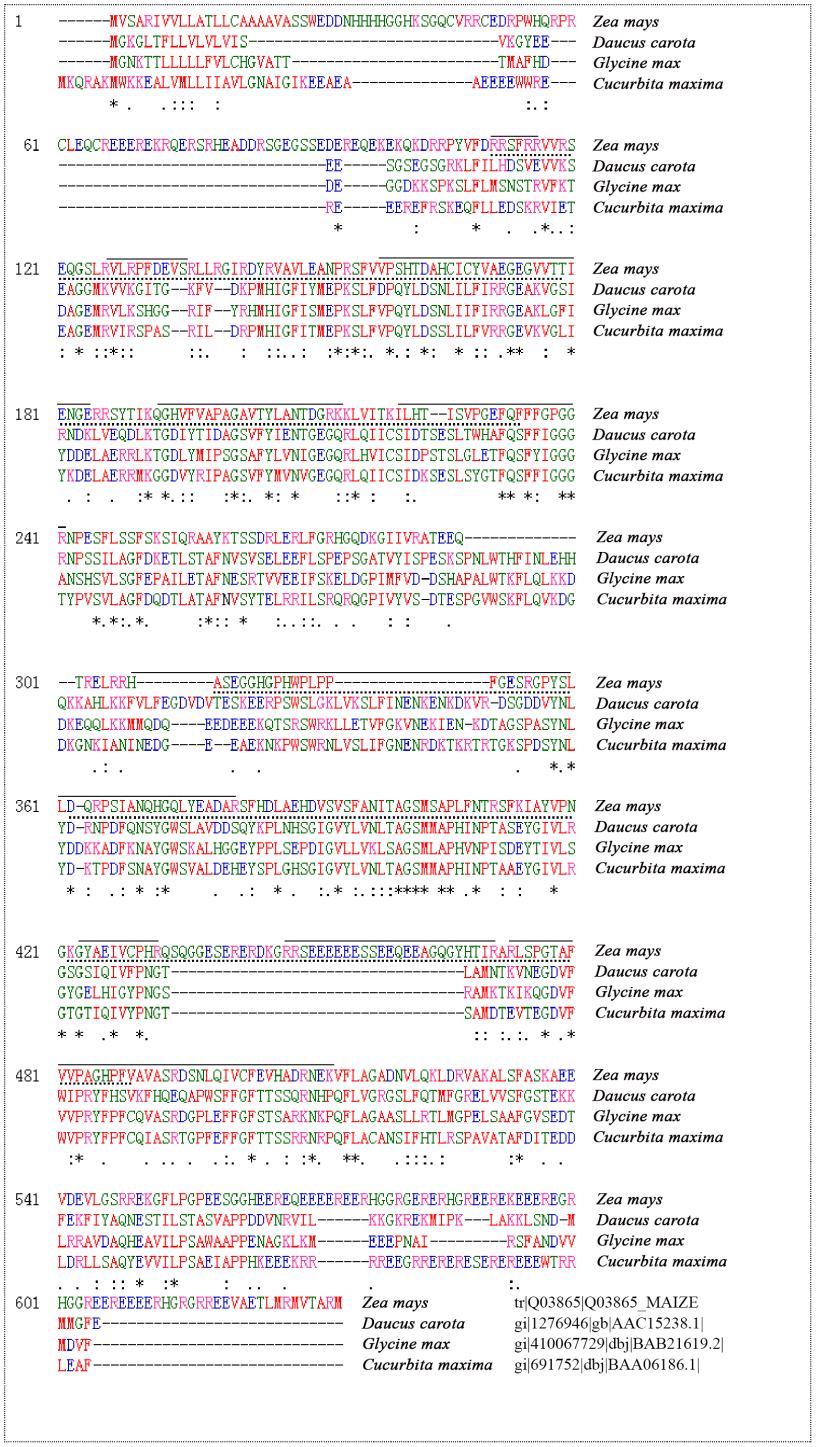
ClustalW alignment of vicilin sequences from *Zea mays*, *Daucus carota*, *Glycine max* and *Cucurbita maxima*. Asterisks (*) under sequences indicate identically conserved residues while double dots (:) indicate conserved substitutions, and singe dots (·) indicate semi-conserved substitutions. The two Cupin1 domains in maize vicilin are indicated with dotted lines under the sequences. The amino acid sequences matched by MS/MS identification were indicated with solid lines above the sequences.

By phenol extraction, the smear region of vicilins was most striking in the top right edge of 2-DE image of maize embryo (**Figure 2**). These vicilins represented at least 20% of total embryo proteins, estimated on their band intensities in 1-D gel. MS/MS analysis (**Table 1**) and sequence alignment by ClustalW2 (http://www.ebi.ac.uk/Tools/msa/clustalw2/) (**Figure 4**) confirmed the vicilins identity of these spots. Maize vicilins contain two typical Cupin domains and share 79% homology with soybean vicilins. Obviously, maize vicilins consist of several major isoforms with similar sizes but different pIs (6.1–6.7), consistent with a previous study that vicilin from maize flour is composed of a string of six spots in 2-D gel, all of them allergenic [1]. Probably, this phenomenon resulted from three causes: (a) maize vicilin is encoded by a single but polymorphic *Glb1* gene [7]–[8], which may contribute the subtle difference of the gene products (protein isoforms); (b) vicilin is a glycoprotein [17] and exists as a heterogeneous mixture after glycosylation; and (c) proteolytic modification occurred during vicilin synthesis also contributes the heterogeneity in vicilin species [18]–[19].

**Table 1:** MS/MS identification of maize embryo proteins of interest.

Spot	Protein/Organism	Uniprot accession	pI/Mr	Mascot Score	Matched peptides	Coverage (%)	Molecular function
1	Globulin-1 S allele precursor/Maize	B6UGJ0	6.16/50.3	204	K.LLAFGADEEQQVDR.V R.FTHELLEDAVGNYR.V	6	Nutrient reservoir activity
2	Globulin-1 S allele precursor/Maize	B6UGJ0	6.16/50.3	179	K.LLAFGADEEQQVDR.V R.FTHELLEDAVGNYR.V	6	Nutrient reservoir activity
3	Globulin-1 S allele precursor/Maize	B6UGJ0	6.16/50.3	301	R.LLDMDVGLANIAR.G K.LLAFGADEEQQVDR.V R.FTHELLEDAVGNYR.V R.FEEFFPIGGESPESFLSVFSDDVIQASFNTR.R	15	Nutrient reservoir activity
4	Globulin-1 S allele precursor/Maize	B6UGJ0	6.16/50.3	140	K.LLAFGADEEQQVDR.V R.FTHELLEDAVGNYR.V	6	Nutrient reservoir activity
5	Globulin-1 S allele precursor/Maize	B6UGJ0	6.16/50.3	284	K.LLAFGADEEQQVDR.V R.FTHELLEDAVGNYR.V R.FEEFFPIGGESPESFLSVFSDDVIQASFNTR.R	13	Nutrient reservoir activity
6	TPA: malate synthase, glyoxysomal/Maize	P49081	6.25/62.2	284	R.IWNGVFQR.A R.VQNWQWLR.H R.DALDFVAGLQR.E R.AGQGAGFGPFFYLPK.M R.AGHDGTWAAHPGLIPAIR.E R.ATVLVETLPAVFQMNEILHELR.E	14	Malate synthase activity
7–9	Unknown protein	-	-	-	Failed to be identified by MS/MS analysis	-	-
10	Vicilin/Maize	Q03865	6.23/66.6	137	R.GPYSLLDQRPSIANQHGQLYEADAR.S R.DSNLQIVCFEVHADRNEK.V	7	Nutrient reservoir activity
11	Vicilin/Maize	Q03865	6.23/66.6	117	R.LSPGTAFVVPAGHPFVAVASR.D R.HASEGGHGPHWPLPPFGESR.G R.GPYSLLDQRPSIANQHGQLYEADAR.S	11	Nutrient reservoir activity
12	Vicilin/Maize	Q03865	6.23/66.6	1014	R.RPYVFDR.R R.VLRPFDEVSR.L R.VAVLEANPR.S K.QGHVFVAPAGAVTYLANTDGR.K K.ILHTISVPGEFQFFFGPGGR.N K.GYAEIVCPHR.Q R.GPYSLLDQRPSIANQHGQLYEADAR.S R.RSEEEEEESSEEQEEAGQGYHTIR.A R.LSPGTAFVVPAGHPFVAVASRDSNLQIVCFEVHADRNEK.V	28	Nutrient reservoir activity
13	Vicilin/Maize	Q03865	6.23/66.6	931	R.RPYVFDR.R R.VLRPFDEVSR.L R.VAVLEANPR.S K.ILHTISVPGEFQFFFGPGGR.N R.HASEGGHGPHWPLPPFGESRGPYSLLDQRPSIANQHGQLYEADAR.S K.GYAEIVCPHR.Q R.RSEEEEEESSEEQEEAGQGYHTIR.A R.LSPGTAFVVPAGHPFVAVASR.D	25	Nutrient reservoir activity
14	Vicilin/Maize	Q03865	6.23/66.6	759	R.RPYVFDR.R R.VLRPFDEVSR.L R.SFVVPSHTDAHCICYVAEGEGVVTTIENGER.R K.ILHTISVPGEFQFFFGPGGR.N R.HASEGGHGPHWPLPPFGESRGPYSLLDQRPSIANQHGQLYEADAR.S R.RSEEEEEESSEEQEEAGQGYHTIRLSPGTAFVVPAGHPFVAVASRDSNLQIVCFEVHADR.N	29	Nutrient reservoir activity
15	Vicilin/Maize	Q03865	6.23/66.6	1068	R.RPYVFDR.R R.VLRPFDEVSR.L R.SFVVPSHTDAHCICYVAEGEGVVTTIENGER.R K.QGHVFVAPAGAVTYLANTDGRK.K K.ILHTISVPGEFQFFFGPGGR.N R.HASEGGHGPHWPLPPFGESRGPYSLLDQRPSIANQHGQLYEADAR.S R.RSEEEEEESSEEQEEAGQGYHTIR.A R.LSPGTAFVVPAGHPFVAVASR.D R.DSNLQIVCFEVHADRNEK.V	34	Nutrient reservoir activity
16	Vicilin/Maize	Q03865	6.23/66.6	1164	R.RPYVFDR.R R.VLRPFDEVSR.L R.SFVVPSHTDAHCICYVAEGEGVVTTIENGER.R K.QGHVFVAPAGAVTYLANTDGRK.K K.ILHTISVPGEFQFFFGPGGR.N R.HASEGGHGPHWPLPPFGESRGPYSLLDQRPSIANQHGQLYEADAR.S R.RSEEEEEESSEEQEEAGQGYHTIR.A R.LSPGTAFVVPAGHPFVAVASR.D R.DSNLQIVCFEVHADRNEK.V	34	Nutrient reservoir activity
17	Vicilin/Maize	Q03865	6.23/66.6	1180	K.QGHVFVAPAGAVTYLANTDGR.K R.VLRPFDEVSR.L K.ILHTISVPGEFQFFFGPGGR.N R.RPYVFDR.R R.GPYSLLDQRPSIANQHGQLYEADAR.S R.RSEEEEEESSEEQEEAGQGYHTIR.A R.LSPGTAFVVPAGHPFVAVASRDSNLQIVCFEVHADRNEK.V	31	Nutrient reservoir activity

The selectivity, efficiency and reproducibility of CAPE depletion of maize vicilins was evaluated using 2-DE (**Figure 2**). After vicilin depletion, 665 (±5) CBB-stained spots were detected in maize embryo extract, compared to 628 (±6) spots without the depletion. In particular, spots 1–17 (**Figure 2**) were selectively removed or newly detected and other representative 12 spots (spots 18–29, **Figure S1**) were enriched in abundance to an extent after CAPE. Moreover, CAPE was highly reproducible based on the 2-DE images from three independent experiments (**Figure S1**).

The 17 spots mentioned above were chosen from the depletion and control gels for MS/MS identification (**Table 1**). The spots lacked in the depletion gel were almost vicilins, indicating a highly selective depletion of CAPE for maize vicilins. Two smaller (spot 10 and 11) and two bigger vicilins (spot 12 and 13) were also removed by CAPE. After vicilin depletion, several low-abundance proteins (**Figure 2**, the enlarged region C and D) were detectable in the places originally taken by vicilins, including globulin-1 S allele precursor (spots 1–5) and TPA: malate synthase (spot 6), which had 42% and 53% identity with maize vicilin, respectively, according to BLAST analysis (http://www.uniprot.org/blast/).

**Figure 5:**
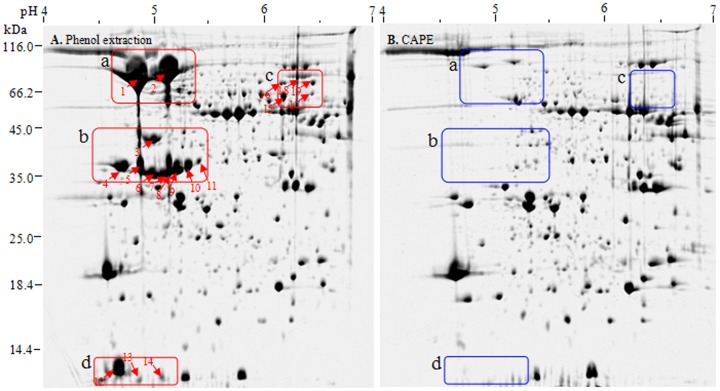
CAPE depletion of abundant storage proteins in soybean embryo extracts. **A**, phenol extraction (control); **B**, CAPE. Proteins (600 µg) were separated by IEF with 11-cm linear pH 4–7 IPG strips and then by SDS-PAGE (13.5% gel). The red and blue rectangle regions indicate before and after vicilin depletion.

The utility of CAPE for dicot seeds was evaluated using soybean seeds. 2-DE analysis showed that CAPE was able to selectively deplete19 abundant protein spots from soybean embryo extracts. These spots were concentrated in four regions of 2-DE image (**Figure 5, a–d**). Recently, a fractionation technique based on 10 mM Ca^+^ precipitation was used to deplete abundant storage proteins from soybean seeds [10]. Estimated on protein sizes and pIs, the depleted protein spots between CAPE (this study) and Ca^+^ fractionation precipitation [10] are almost the same, especially in the regions **a** and **b**. However, the quality of 2-DE image of soybean embryos after CAPE depletion was superior to that obtained by Ca^+^ fractionation precipitation. Therefore, CAPE was able to deplete abundant storage proteins from both monocot and dicot embryo extracts.

Obviously, CAPE improved proteome profiling of embryos in two ways: first, it selectively depletes vicilins (or abundant storage proteins) and allows the detection of low-abundance spots which were masked in 2-DE gels; second, it allows greater protein loads and meantime reduces spot ‘tailing’ in 2-DE, thus having potential to resolve more spots, especially using sensitive proteomic approaches (e.g. iTRAQ, DIGE).

### Rationale of CAPE to selectively deplete abundant storage proteins

The novelty of CAPE is selectively depletion of vicilins or abundant storage proteins from embryo extracts. So, the rationale behind CAPE was explored.

**Figure 6:**
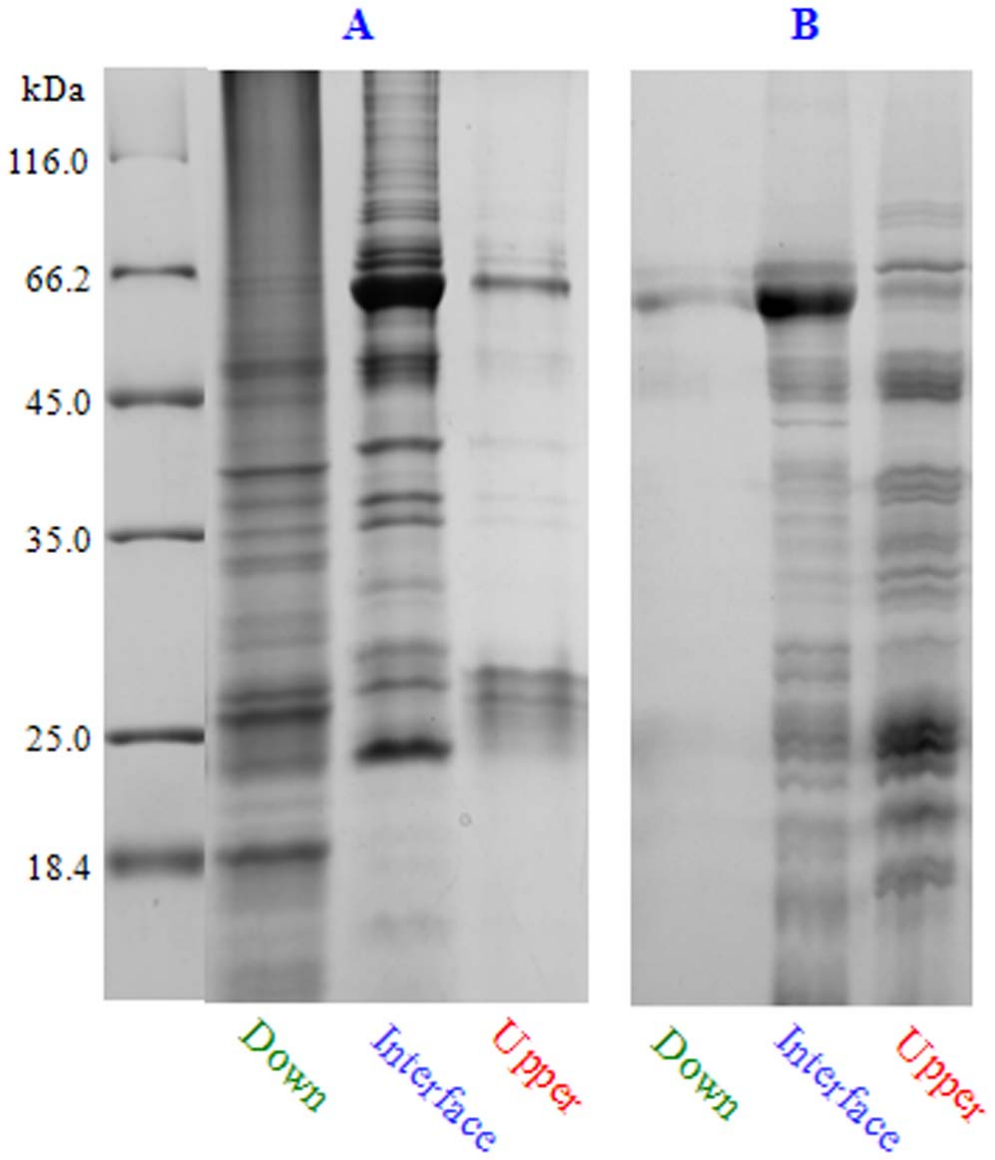
The effect of chloroform on the behavior of maize vicilins during phase separation. After phase separation, the proteins from upper phase, interface and down phase were analyzed using SDS-PAGE. **A**, CAPE. **B**, chloroform extraction.

We first examined the performance of maize vicilins during phase separation in CAPE. The proteins recovered from upper phase, interface and down phase of the chloroform/phenol/aqueous mixture were compared using SDS-PAGE (**Figure 6**). As a result, the overwhelming majority of maize vicilins were found to aggregate in the interface, while only a small amount remained in the upper phase and no vicilin band was detected by CBB staining in the down phase. Obviously, compared to other embryo proteins, maize vicilins were more susceptible to chloroform denaturing and prone to aggregate. These strong aggregates were not be resolubilized in the chloroform/phenol phase of CAPE. However, this was open to question: why were vicilins (or specific abundant storage proteins) depleted selectively by CAPE? Probably, the rationale behind CAPE was due to the specific interaction of chloroform and proteins, depending on the physicochemical property, spatial structure, especially amino acid sequence of proteins.

Likewise, a chloroform/aqueous buffer mixture, maize vicilins aggregated in the interface (**Figure 6**). As phenol extraction and other protein extraction protocols, CAPE inevitably resulted in the loss of a small amount of proteins in the interface and the upper phase.

Chloroform is a commonly used protein denaturing agent. Due to highly compatible with phenol and alcohol, chloroform is routinely used in molecular biology to separate proteins from DNA or RNA [20]–[21]. Moreover, chloroform is an effective organic solvent to extraction membrane protein [22]–[24]. The CAPE method reported here extends the application of chloroform, especially phenol extraction, in seed protein analysis. In addition, our results showed that vicilins are prone to aggregate in the interface during phase separation in CAPE; therefore, CAPE also provides a simple method to enrich and partially purify vicilins (or specific abundant storage proteins) from embryo extracts for downstream studies.

In conclusion, the present work reports a novel CAPE method for high-abundance storage proteins depletion from both monocot and dicot embryo extracts. The method is simple, low cost and compatible with proteomic analysis. It can significantly improve embryo proteome profiling and extends the application of chloroform and phenol extraction in proteomics.

## Supporting Information

Figure S1 **Depletion of maize vicilins by CAPE.** A and B represented two groups of independent experiments. Maize embryo proteins were separated by IEF with 11-cm linear pH 4–7 IPG strips and then by SDS-PAGE. C, graphic column of relative spot volume. P, phenol extraction. C, CAPE.
(TIF)

## References

[1] Fasoli E, Pastorello EA, Farioli L, Scibilia J, Aldini G, et al (2009) Searching for allergens in maize kernels via proteomic tools. J Proteomics 72: 501–510. 19367736 10.1016/j.jprot.2009.01.013

[2] Colmsee C, Mascher M, Czauderna T, Hartmann A, Schlüter U, et al (2012) OPTIMAS-DW: A comprehensive transcriptomics, metabolomics, ionomics, proteomics and phenomics data resource for maize. BMC Plant Biol 12: 245. 23272737 10.1186/1471-2229-12-245 PMC3577462

[3] Shewry PR, Halford NG (2002) Cereal seed storage proteins: structures, properties and role in grain utilization. J Exp Bot 53: 947–958. 11912237 10.1093/jexbot/53.370.947

[4] Shewry PR, Napier JA, Tatham AS (1995) Seed storage proteins: structures and biosynthesis. Plant Cell 7: 945–956. 7640527 10.1105/tpc.7.7.945 PMC160892

[5] Gibbs PE, Strongin KB, McPherson A (1989) Evolution of legume seed storage proteins—a domain common to legumins and vicilins is duplicated in vicilins. Mol Biol 6: 614–623. 10.1093/oxfordjournals.molbev.a040575 2488475

[6] Astwood JD, Silvanovich A, Bannon GA (2002) Vicilins a case study in allergen pedigrees. J Allergy Clin Immunol 110: 26–27. 12110813 10.1067/mai.2002.125690

[7] Belanger FC, Kriz AL (1989) Molecular characterization of the major maize embryo globulin encoded by the *Glbl* gene. Plant Physiol 91: 636–643. 16667080 10.1104/pp.91.2.636 PMC1062048

[8] Belanger FC, Kriz AL (1991) Molecular basis for allelic polymorphism of the maize *Globulin-1* gene. Genetics 129: 863–872. 1752424 10.1093/genetics/129.3.863 PMC1204753

[9] Kriz AL, Wallace MS, Paiva R (1990) Globulin gene expression in embryos of maize viviparous mutants: Evidence for regulation of the *Gibl* gene by ABA. Plant Physiol 92: 538–542. 16667311 10.1104/pp.92.2.538 PMC1062327

[10] Krishnan HB, Oehrle NW, Natarajan SS (2009) A rapid and simple procedure for the depletion of abundant storage proteins from legume seeds to advance proteome analysis: A case study using *Glycine max* . Proteomics 9: 3174–88. 19526550 10.1002/pmic.200800875

[11] Kim YJ, Lee HM, Wang Y, Wu J, Kim SG, et al (2013) Depletion of abundant plant RuBisCO protein using the protamine sulfate precipitation method. Proteomics 13: 2176–2179. 23576416 10.1002/pmic.201200555

[12] Wu XL, Xiong EH, An SF, Gong FP, Wang W (2012) Sequential extraction results in improved proteome profiling of medicinal plant *Pinellia ternata* tubers, which contain large amounts of high-abundance proteins. PLoS One 7: e50497. 23185632 10.1371/journal.pone.0050497 PMC3502364

[13] Liu B, Qiu FH, Courtney V, Xu Y, Zhao MZ, et al (2011) Evaluation of three high abundance protein depletion kits for umbilical cord serum proteomics. Proteome Science 9: 24. 21554704 10.1186/1477-5956-9-24 PMC3105942

[14] Bellei E, Bergamini S, Monari E, Fantoni LI, Cuoghi A, et al (2011) High-abundance proteins depletion for serum proteomic analysis: concomitant removal of non-targeted proteins. Amino Acids 40: 145–156. 20495836 10.1007/s00726-010-0628-x

[15] Wu XL, Xiong EH, Wang W, Scali M, Cresti M (2014) Universal sample preparation integrating trichloroacetic acid/acetone precipitation and phenol extraction for crop proteomics. Nat Protoc 9: 362–374. 24434803 10.1038/nprot.2014.022

[16] Wu XL, Gong FP, Wang W (2014) Protein extraction from plant tissues for two-dimensional gel electrophoresis and its application in proteomic analysis. Proteomics 14: 645–658. 24395710 10.1002/pmic.201300239

[17] Gerlach JQ, Bhavanandan VP, Haynes PA, Joshi L (2009) Partial characterization of a vicilin-like glycoprotein from seeds of flowering tobacco (*Nicotiana sylvestris*). J Bot Doi:10.1155/2009/560394.

[18] Higgins TJ, Newbigin EJ, Spencer D, Llewellyn DJ, Craig S (1988) The sequence of a pea vicilin gene and its expression in transgenic tobacco plants. Plant Mol Biol 11: 683–695. 24272502 10.1007/BF00017468

[19] Fukuda T, Prak K, Fujioka M, Maruyama N, Utsumi S (2007) Physicochemical properties of native adzuki bean (*Vigna angularis*) 7S globulin and the molecular cloning of its cDNA isoforms. J Agric Food Chem 55: 3667–3674. 17417864 10.1021/jf063205l

[20] Sambrook J, Russell DW (2006) Purification of nucleic acids by extraction with phenol: chloroform. CSH Protoc doi:10.1101/pdb.prot4455 10.1101/pdb.prot4455 22485786

[21] Chomczynski P, Sacchi N (2006) Single-step method of RNA isolation by acid guanidinium thiocyanate-phenol-chloroform extraction: Twenty-something years on. Nat Protoc 1: 581–585. 17406285 10.1038/nprot.2006.83

[22] Mirza SP, Halligan BD, Greene AS, Olivier M (2007) Improved method for the analysis of membrane proteins by mass spectrometry. Physiol Genomics 30: 89–94. 17341690 10.1152/physiolgenomics.00279.2006 PMC2814522

[23] DI Girolamo F, Ponzi M, Crescenzi M, Alessandroni J, Guadagni F (2010) A simple and effective method to analyze membrane proteins by SDS-PAGE and MALDI mass spectrometry. Anticancer Res 30: 1121–1129. 20530417

[24] Reigada R (2014) Electroporation of heterogeneous lipid membranes. Biochim Biophys Acta 1838: 814–821. 24144543 10.1016/j.bbamem.2013.10.008

