# Supplementary figures and images for: Chloroform-Assisted Phenol Extraction Improving Proteome Profiling of Maize Embryos through Selective Depletion of High-Abundance Storage Proteins
Source: PLoS One. 2014 Nov 11;9(11):e112724. doi: 10.1371/journal.pone.0112724 (PMC4227863; doi:10.1371/journal.pone.0112724)

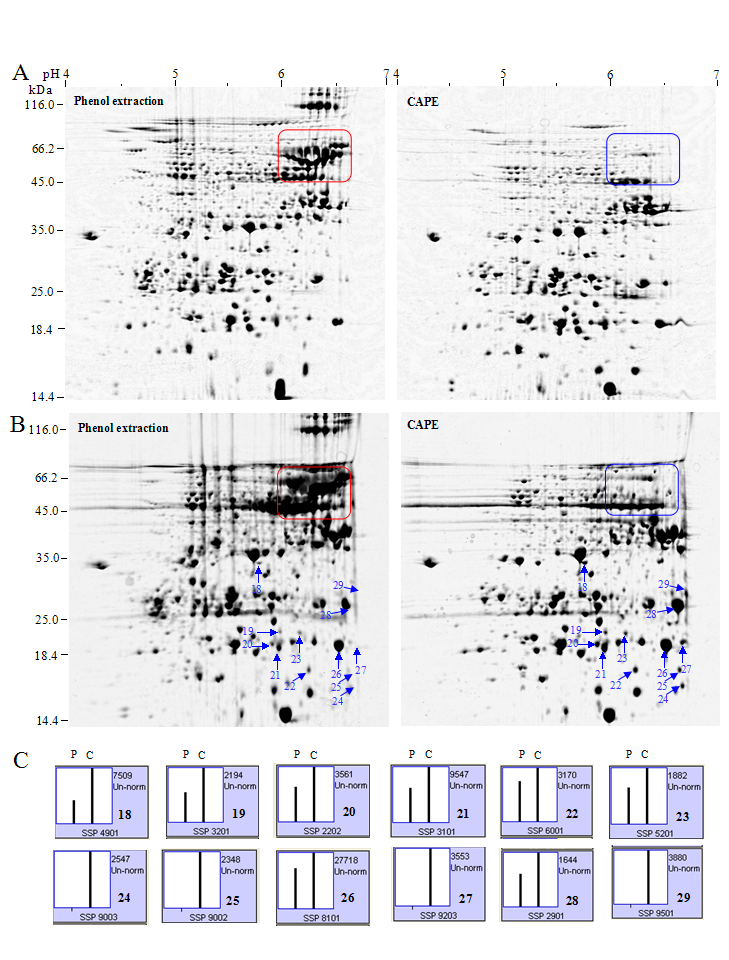

Supplement: Figure S1 — Depletion of maize vicilins by CAPE. A and B represented two groups of independent experiments. Maize embryo proteins were separated by IEF with 11-cm linear pH 4–7 IPG strips and then by SDS-PAGE. C, graphic column of relative spot volume. P, phenol extraction. C, CAPE. (TIF) [file pone.0112724.s001.tif]
